# Enhancing comprehensive in primary care: results of a cross-sectional survey of primary care social workers in Ontario, Canada

**DOI:** 10.1186/s12875-026-03226-4

**Published:** 2026-02-27

**Authors:** Rachelle Ashcroft, Nele Feryn, Simon Lam, Amina Hussain, Rumia Owaisi, Peter Sheffield, Deepy Sur, Jennifer Rayner, Catherine Donnelly, Keith Adamson, Judith Belle Brown

**Affiliations:** 1 Factor-Inwentash Faculty of Social Work, University of Toronto, 246 Bloor Street West, Toronto, ON M5S 1V4 Canada; 2 Ghent University, Ghent, Belgium; 3 King’s School of Social Work, Western University, London, ON Canada; 4 Ontario College of Family Physicians, Toronto, ON Canada; 5 Alliance for Healthier Communities, Toronto, ON Canada; 6 School of Rehabilitation Therapy, Queen’s University, Kingston, ON Canada; 7 Department of Family Medicine, Western University, London, ON Canada

**Keywords:** Social work, Primary care, Scope of practice, Professional role, Survey

## Abstract

**Background:**

Social workers are key members of team-based, primary care and offer a wide range of health, mental health, and social well-being services to diverse communities. This study sought to clarify what is known about social work’s scope of practice in primary care settings across Ontario, Canada.

**Methods:**

A cross-sectional online survey was disseminated to primary care social workers across Ontario, Canada. The survey consisted of 45 items and included closed-ended and open-ended questions. Survey domains included practice characteristics, background on role, primary care context, structure of practice, clinical activities, and demographics.

**Results:**

*N* = 159 social workers across Ontario healthcare regions and primary care settings completed the survey. Social workers reported working with individuals across the life span, and engaging in a range of practice activities primarily related to mental health and counselling. Frequent interactions were reported with other healthcare professionals including physicians and nurses. However, increased collaboration was recommended to improve patient care. Social workers provided insight into their caseloads, referral systems, wait times, and follow-up procedures. They highlighted how time spent with patients was context-dependent, and that currently used performance metrics may not always adequately capture the nuances of their work.

**Conclusions:**

Social workers collaborate with primary care teams to attend to a variety of community and patient needs, reflecting expertise in delivering health, mental health, and social well-being services. There is a need to develop best practices and guidelines for social work practice that optimizes care to communities while also promoting provider well-being.

**Supplementary Information:**

The online version contains supplementary material available at 10.1186/s12875-026-03226-4.

## Background

Expanding interprofessional primary care teams has been a growing focus in Canada, the United States, and other international settings in recent years [1–5]. Primary care acts as the first point of contact for patients and serves as the cornerstone of healthcare systems worldwide [6, 7]. *Team-based* primary care broadens the scope of health and mental health services by integrating professionals from various disciplines to collaborate with family physicians and/or nurse practitioners [8, 9]. While common providers in primary care teams include family physicians, nurse practitioners, nurses, social workers, pharmacists, physiotherapists, dietitians, and psychologists, the types and numbers of providers can differ from one team to another [8–10], as team size and composition is determined based on the health service needs of the local population [11]. Enhancing comprehensiveness of care, or the range of services available to patients, is a foundational attribute and key benefit of team-based primary care [8–10]. With the expansion of interprofessional team-based care [5], the number of social workers in primary care is projected to increase substantively [12, 13] in part due to the rising demands for mental health care services as well as the increasing social complexity of primary care patients due to conditions related to the social determinants of health [12–14].

Social work is a practice-based profession that addresses a wide range of needs for individuals, families, and communities, with an emphasis on improving health, mental health, and social well-being [15]. Clinical social work is one of the largest health, mental health, and social service professions in Canada, the United States, and worldwide [13, 15–17]. The Canadian Institute for Health Information [18] reports approximately 52,823 registered social workers across Canada, while the Bureau of Labor Statistics states that over 715,000 social workers are employed across the United States [12]. Social workers are employed in various settings, including healthcare, mental health and addictions, child welfare, education, and many other specialized fields [15]. Driven by policy reforms in the early 2000s promoting interprofessional team-based care, an increasing number of social workers have been integrated into primary care settings in countries such as Canada, the United States, and elsewhere [2, 10, 13, 17]. Current initiatives are further striving to strengthen and expand access to team-based care resulting from the critical and ongoing shortage of family physicians in primary care [1, 4, 5]. Inclusion of social workers in primary care improves team capacity for delivering foundational care and addressing patient needs that may otherwise become deprioritized [19]. The biopsychosocial approach, person-in-context philosophy, and clinical expertise of social workers align well with the goals of primary care [13, 17, 19].

Social work practice in primary care is diverse, encompassing individual, family, and group therapy or counseling; case management; crisis services; comprehensive risk assessment; health promotion and patient education; system navigation and resource allocation; social prescription; community organization; program and policy development; advocacy; conducting ongoing case management; and providing general support related to the range of health and mental health conditions seen in primary care [19]. They also play a key role in facilitating team processes promoting strong workplace dynamics by fostering patient-provider relationships, engaging in team building, and contributing to the education and training of other healthcare providers [10, 17, 19, 20]. Social work services may be delivered in-person, virtually, and across various settings [9, 19, 21], although the typical length of social worker appointments in primary care settings is not well-defined [19, 22]. Home visits may also be conducted for homebound patients, particularly in cases of palliative and end-of-life care or other complex medical and psychosocial situations [19, 23]. Indirect tasks requiring less face-to-face contact are also a component of social work practice in primary care, including such activities like care coordination and documentation [10, 19, 22]. Outside of direct patient care, social workers often engage in community development, outreach, health promotion initiatives, and a range of formal and informal leadership roles [10, 20, 23, 24].

Research on the role of social workers in primary care is needed to inform the current growth and expansion of clinical social work services in primary care [10, 19, 23]. Moreover, there is a need to improve organizational structures and processes [25, 26] that promote collaboration, integration, and practicing at full scope for social workers in primary care teams to optimize the quality of patient care. The overarching research question guiding this study is as follows: What is the nature of social work practice in primary care settings in Ontario, Canada? The specific objectives are to (i) determine how social workers in primary care contribute to patient care, (ii) identify how social workers collaborate within the interprofessional primary care team context, and (iii) explain what key structures and processes guide social work practice in primary care.

### Setting

The study was administered in Ontario, Canada’s most populous province (16.1 million residents) [27], where dedicated provincial policy reforms since 2005 strengthened interprofessional team-based primary care [7], making it an ideal setting in which to conduct the study. Policy reform includes the introduction of various payment schemes, including rostered patients which consists of patients formally signing up to receive care from a designated primary care physician [28]. On the other hand, non-rostered patients are those who do not have a designated primary care physician. In Ontario, there are various interprofessional primary care team models that include social workers such as Family Health Teams (FHTs) which serve 25% of the population, Community Health Centres (CHCs) which offer care to approximately 2% of the population and focuses on clients with more complex health and social care needs, Nurse Practitioner Led Clinics (NPLCs) which embeds nursing leadership within team-based care, and Aboriginal Health Access Centres (AHACs) which offers community-led, wholistic care to Indigenous people [11, 29–31].

## Methods

### Design

This study used a descriptive cross-sectional online survey design distributed to social workers employed in primary care in the province of Ontario, Canada. Such surveys are effective for gathering large quantities of data from professionals in an efficient manner [32]. The online web-based survey was developed for this study using Qualtrics (Qualtrics. Provo, UT, USA. 2013) and was informed by a previous survey designed to examine social work practice in primary care teams in Ontario, Canada [10] which was inspired by previous research examining occupational therapy [33] and various providers in primary care teams [9] in the province of Ontario, Canada. The survey was developed in collaboration with our partner organizations - The Association of Family Health Teams of Ontario (AFHTO), the Alliance for Health Communities (Alliance), and the Ontario Association of Social Workers (OASW). All members of the research team reviewed and revised survey questions to ensure clarity.

The final English web-based survey included 45-items, 41 closed-ended and 4 open-ended, and took an average of 15 min to complete. Approximate survey domains included (1) practice characteristics, (2) role description, (3) primary care context, (4) structure of practice, (5) improving the structure of practice, (6) clinical activities, (7) leadership, and (8) demographics. Prior to implementation, we conducted a cognitive interview to trial and help improve the survey tool [32]. Cognitive interviews are used in health care research to improve survey design [32]. Cognitive interviewing means that an informed respondent with an expertise in primary care social work completed the survey in the presence of a research assistant (AH) and was asked to think out loud as she went through the survey [34]. See supplemental file 1 for a copy of the survey. Participants were required to provide informed consent prior to commencement of the survey, and all participants provided consent. Research ethics approval was obtained at the University of Toronto in Toronto, Canada (REB#41200).

### Sample, data collection, and data analysis

A convenience sample of social workers employed in team-based primary care in Ontario was obtained. Although social workers are employed across many primary care settings in Ontario, the absolute numbers of social workers are unknown. Recruitment methods were mainly completed using direct emails to social workers in primary care settings (FHTs, CHCs, NPLCs, AHACs) via our three community partners - AFHTO, Alliance, and OASW – that helped to reach most social workers employed in primary care. As well, social media (i.e., X/Twitter) was used to share information for recruitment. Participation in the survey was voluntary and without recruitment incentive. This manuscript presents the results of all survey domains, with the exception of leadership because these data were previously reported [24]. Descriptive statistics on the close-ended questions were generated using Qualtrics software, and the four open-ended questions were analyzed using thematic analysis. One member of the research team identified and highlighted themes in the open-ended responses which was reviewed by a senior member of the research team. In addition, a small group of researchers met to discuss the themes on a regular basis and made decisions when there are disagreements. Qualitative data was used in a way to support the quantitative results. This English-language survey was administered to participants via Qualtrics between April 4, 2022, and November 9, 2022.

## Results

### Respondent demographics

A total of 159 social workers employed in primary care participated in the survey. Respondents worked in primary care settings across the five Ontario Health Regions: West (37%), East (20%), Central (16%), Toronto (14%), North (12%), and unknown (1%). A range of primary care settings were reported including FHTs (52%), CHCs (35%), NPLCs (6%), and other (7%). “Social worker” was the most frequently reported formal job title (74%), followed by “mental health counsellor” (23%), and “registered psychotherapist” (9%). Respondents also indicated “other” roles (11%) that included outreach support worker, case manager, and child and family therapist. The majority of respondents (77%) reported working full-time within primary care, for a single employer. Table 1 illustrates the demographics of the respondents.

**Table 1:** Respondent demographics

	Number of Participants (%)
Years in Practice as a Social Worker	
Less than 1 year 1 to 5 years 6 to 10 years 11 to 15 years 16 to 20 years Over 20 years No response	3 (2%) 28 (18%) 25 (16%) 34 (21%) 20 (13%) 41 (26%) 8 (5%)
Years of Working in Current Position	
Less than 1 year 1 to 2 years 3 to 4 years 5 to 6 years 7 to 8 years Over 8 years No response	27 (17%) 27 (17%) 37 (23%) 14 (9%) 9 (6%) 37 (23%) 8 (5%)
Educational background*	
Master of Social Work Bachelor of Social Work Social Service Worker Diploma Other No response	115 (72%) 40 (25%) 11 (7%) 18 (11%) 8 (5%)
Age (years)	
18 to 25 26 to 40 41 to 55 56 to 70 Prefer not to answer No response	2 (1%) 44 (28%) 56 (35%) 25 (16%) 3 (2%) 29 (18%)
Racial or cultural groups*	
White First Nations South Asian Black Chinese Métis Other Prefer not to answer No response	102 (64%) 5 (3%) 5 (3%) 5 (3%) 4 (3%) 3 (2%) 10 (6%) 5 (3%) 29 (18%)
Gender	
Female Male Non-binary Two-Spirit Other No response	108 (68%) 18 (11%) 1 (1%) 1 (1%) 2 (1%) 29 (18%)

*Responses in these categories exceed more than 100% because respondents had option to select all that apply

All respondents indicated having an educational background in social work, with 72% holding a Master of Social Work and 25% possessing a Bachelor of Social Work. For 74% of respondents, a Master’s degree in social work was the minimum required educational background for their currently held employment position, while 23% reported that their currently held employment position required a bachelor’s degree. A Master’s in a related field such as counselling, or psychology was mentioned as a minimum requirement by the remaining 9% of employment positions held by respondents. Registration with the Ontario College of Social Workers and Social Service Workers was noted as a requirement for 92% of respondents.

### Collaboration within team based care

In terms of collaborating with other social workers, respondents noted that the distribution of social workers across primary care settings varied. Some (13%) respondents stated they were the sole social worker within their primary care organization, and others (47%) reported that they were employed alongside two to three other social workers within the same primary care organization. Nearly a quarter (23%) expressed being a part of a team of four to seven social workers, while the remaining 17% of respondents reported a team of eight or more social workers within the same primary care organization. In terms of being physically co-located within the same primary care workplace setting, 45% of the respondents reported being co-located with two or three other social workers, while 27% of the participants stated that they had no other social workers who were physically co-located in the same setting. Additionally, 17% of the respondents reported being in a workplace where they were co-located with four or five social workers, while 3% worked alongside six or seven other social workers. Being co-located with nine of more social workers was noted by 6% of social workers.

**Fig. 1:**
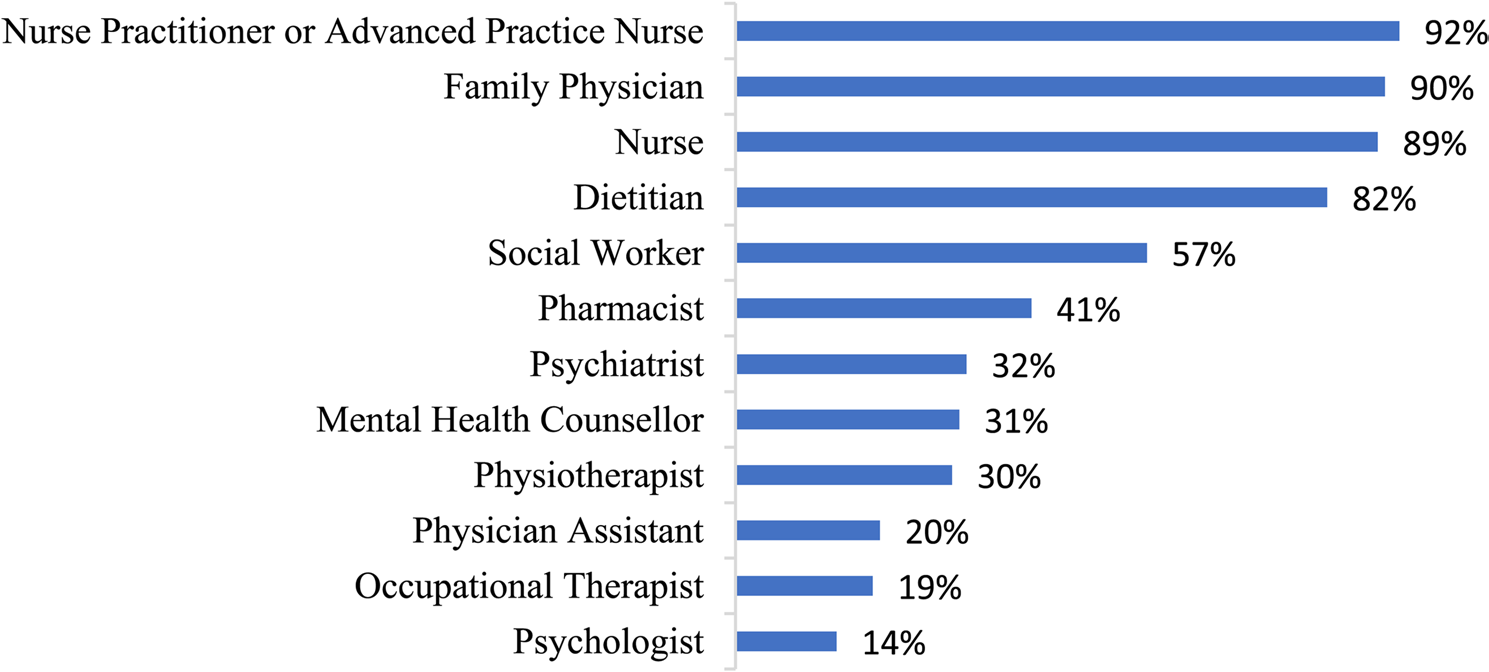
Types of providers that social workers in primary care collaborate with within the immediate primary care team (*n* = 145)

**Fig. 2:**
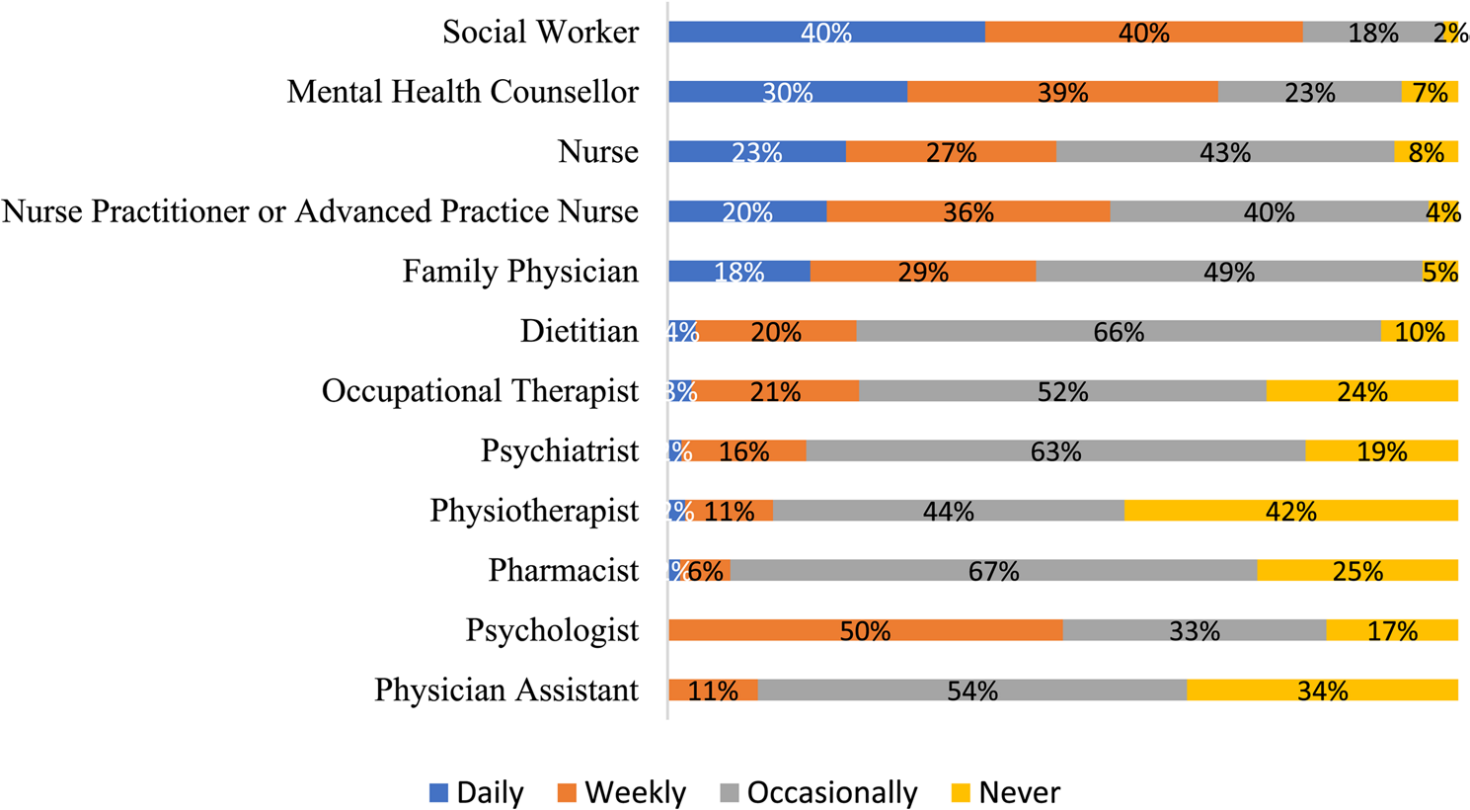
Frequency of communication with other members of the primary care team (*n* = 145)

**Fig. 3:**
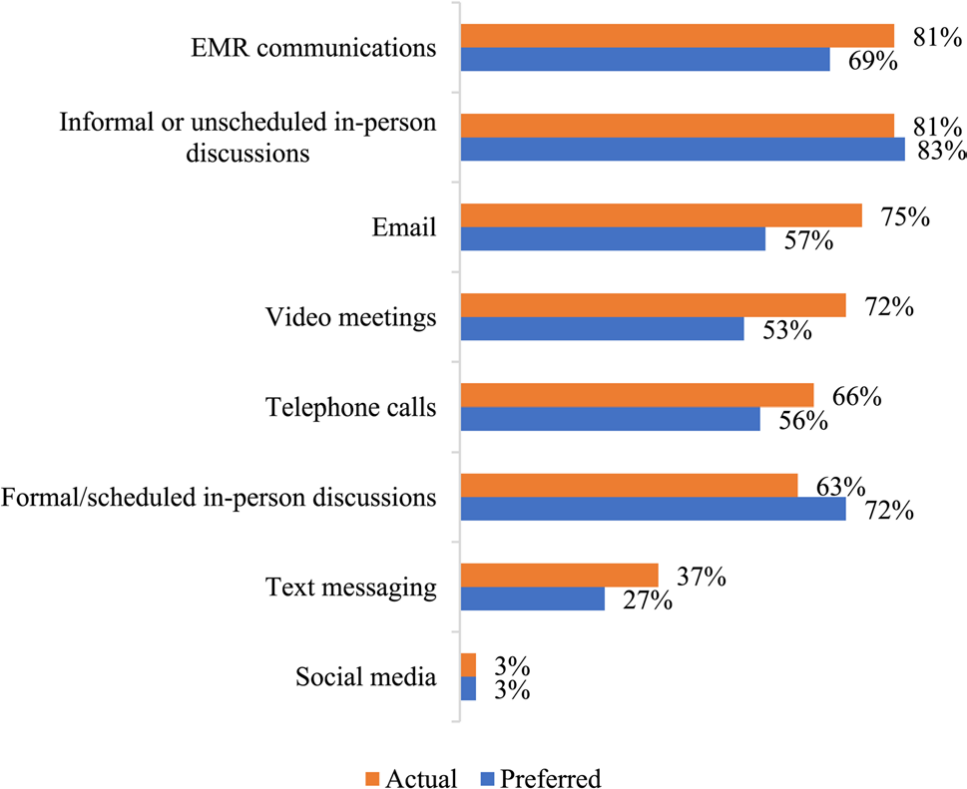
Actual and preferred methods of communication with other members of the primary care team (*n* = 145)

Respondents indicated that social workers collaborated with a broad range of service providers as outlined in Fig. 1, while Fig. 2 highlights the frequency that social workers communicate with other types of service providers within their immediate primary care team. In terms of the actual modality being used for daily interactions between social workers and other members of the primary care team, respondents noted predominantly relying on electronic medical record (EMR) communications (81%), informal, unplanned in-person discussions (81%), email (75%), video meetings (72%), formal in-person discussions (63%), and telephone calls (66%). Less frequently used methods included text messaging (37%) and social media (3%). Some (3%) mentioned other methods such as using Google chats or other types of secure chats. Figure 3 presents results of the current *actual* methods being used to communicate with other members of the primary care team and the *preferred* methods for communicating with other members of the primary care team.

### Caseload

Most respondents (69%) typically saw between 1 and 5 patients in a workday for individual appointments. A quarter of respondents (25%) saw between 6 and 10 patients per day for individual appointments. Lastly, a small percentage (1%) of social workers reported seeing more than 11 patients each day. 76% of respondents indicated that five individual appointments per day is the maximum they recommend for a social worker in primary care, while 14% recommended a maximum of 6 to 10 appointments. Respondents indicated seeing between 16 and 20 (26%) or 21–25 (28%) patients per week. Most respondents (85%) shared that they typically scheduled between 30 and 60 min for a follow-up appointment, and most respondents (85%) also considered this the ideal length for a follow-up appointment.

### Performance metrics

When asked about the adequacy of currently used performance metrics to capture social workers’ contributions to patient care, 26% of respondents reported that existing performance metrics were adequate, while 14% reported being unsure. The remaining 60% did not believe they were adequate and expanded on their thoughts using the open-text option: *“Our health model… uses CBT-based methodology or other evidence-based practices to measure progress and it misses other social determinants of health*,* such as housing and food security. These are factors that determine overall wellness for which CBT or other evidence-based practices do not measure.”* Respondents noted that currently used performance metrics primarily focused on capturing the time spent with clients and inadequately considered the intensity, quality, and complexity of cases: *“It keeps track adequately of how many patients we see face to face*,* but I’m not sure that current measure adequately keep tracks of the work we really do.”*

Many social workers highlighted the value of feedback and notes, discussing how these notes in patient records offered a more comprehensive picture of the care provided in comparison to relying on statistics alone. They also added that indirect ways of working with clients, such as researching sources, developing treatment plans, and documenting interactions in patient records may not be accurately captured by statistics: *“It really only tracks direct service to patients and doesn’t track all of the non-direct ways we work with clients*,* such as researching resources for them*,* putting together treatment plans*,* documenting interactions in their charts or admin time.”* Some respondents mentioned that existing performance metrics adequately captured relevant information: *“It balances all parties involved in and uses standardized evidence-based tools*,* psychometric*,* client reports self-reports*,* statistical clinical observations and analysis.”* In response to an open-text question on what performance metrics were being used to track social workers’ contribution to patient care, EMR patient data was mentioned.

### Wait times and referrals

When it comes to waiting times for urgent referrals, 15% of respondents stated they were able to accommodate same-day appointments. Another 37% indicated a waiting time of one week, while 11% had a waiting time of two weeks. Services for urgent referrals were not offered by 28% of social workers. Conversely, for non-urgent referrals, 32% of social work respondents reported having a waiting time of 5 weeks. Same-day non-urgent availability was indicated by 4% of respondents, 14% had a wait time of one week, and 49% had waiting times ranging from two to four weeks. Over a third (36%) of participants offered care to patients who were not rostered with their primary care clinic, with services provided to non-rostered patients ranging from psychoeducational therapy groups, counseling, and providing outreach work to homeless populations, shelters, and the community. In an open-text box provided for respondents to elaborate on services for non-rostered patients, a respondent noted: *“I provide substance use and psychotherapy services to community members*,* and approximately 95% of my caseload consists of non-rostered patients”.*

Respondents noted that initiating social work services with a new patient mainly occurred by referrals sent by family physicians or nurse practitioners (89%), referrals sent by other members of the interprofessional team (65%), or by way of the patient themself making direct first contact with the social worker (36%). In an open-text box, some respondents indicated that they received referrals from other sources, including sources outside of the immediate primary care team such as from schools, community services, and hospitals. Many respondents (44%) expressed that improvements could be made to the existing referral process to improve access and efficiency of social work services.

### Practice activities of social workers in primary care

Social workers reported working with individuals across the lifespan: 95% stated they worked with adults aged 30–64; 78% mentioned working with young adults aged 19–29; 65% reported supporting older adults aged 65 and older; 42% noted working with youth aged 13–18; and 19% indicated working with children under 13. Individual counselling sessions took up at least half of total working hours for 83% of social workers. Couple and/or family counselling and group work occurred less frequently, with 40% mentioning that it occupied up to a quarter of their working hours, while 60% of respondents indicated never engaging in couple and/or family counselling sessions or group work. Table 2 illustrates the various practice areas social workers dedicate their time to on a daily, weekly, and monthly basis. Respondents were asked to estimate how frequently their social work practice was focused on the practice areas listed in Table 2. Mental health was reported as a main practice area by all respondents, with 97% indicating it as a daily area of focus. In an open-text box, respondents who selected the “Other” category indicated that their frequent practice areas included: trauma, food insecurity, unemployment, intimate partner violence, social isolation, behaviours and stress, work stressors, immigration, social isolation, and management and leadership.

**Table 2:** Percentage of time spent directed to specific types of practice areas

Types of Practice Areas	Daily	Weekly	Month	Rarely	Never
Mental Health	97%	3%	0%	0%	0%
Financial stressors, social assistance	33%	41%	15%	8%	3%
Chronic disease management	24%	33%	17%	17%	8%
Housing insecurity	23%	33%	23%	16%	5%
Parenting issues	22%	29%	28%	16%	6%
Addictions	19%	40%	22%	14%	5%
Children and youth	19%	27%	24%	14%	17%
Grief and bereavement (not palliative care related)	17%	46%	28%	8%	2%
Geriatrics and aging	10%	32%	26%	17%	16%
Legal issues	8%	23%	29%	32%	8%
Trans-specific and gender affirming care	6%	19%	26%	37%	13%
Memory clinic and/or other neurological issues	1%	18%	23%	25%	33%
Palliative care	1%	7%	20%	35%	38%
Other	52%	16%	0%	0%	32%

Table 3 highlights practice activities social workers reported engaging in on daily, weekly, and monthly basis. Respondents were asked to estimate how frequently they engaged in the practice activities listed in Table 3. Most frequent daily activities included documentation (98%) and direct counselling or therapy (95%). General psychosocial assessments were conducted by 47% of social workers daily, while 39% reported doing so on a weekly basis. Over half of social workers (55%) conducted formal assessments using an assessment tool either daily or weekly. Leading psychoeducational groups was a more infrequent activity with 2% engaging in it daily, 26% doing so on a weekly basis, and 34% indicating that they have never led a group. In response to an open-text question asking about types of patient groups offered, those respondents who facilitated psychoeducational therapy groups indicated that cognitive behavioural therapy (CBT) groups were offered on a range of topics. In an open-text box, items noted as daily or weekly in the “Other” category included practice activities pertaining to trauma treatment, memory clinic, completing applications, administrative tasks, attending meetings, and activities related to management and leadership.

**Table 3:** Percentage of time spent engaged in practice activities within previous four weeks

Type of practice activity	Daily	Weekly	Month	Rarely	Never
Documentation	98%	2%	0%	0%	1%
Direct counselling/therapy	95%	3%	1%	0%	2%
General psychosocial assessments	47%	39%	5%	6%	4%
Systems navigation and accessing community resources	35%	38%	16%	8%	3%
Case management	29%	28%	22%	11%	10%
Formal assessments using a tool (e.g., PHQ9)	23%	32%	18%	21%	6%
Providing consultation to healthcare providers WITHIN your team (related to patient care)	20%	41%	26%	10%	3%
Educating and training students	10%	8%	8%	21%	53%
Providing consultation to healthcare providers OUTSIDE of your team (related to patient care)	3%	19%	31%	30%	16%
Leading psychoeducational therapy groups	2%	26%	17%	21%	34%
Other	18%	12%	12%	6%	53%

### Types of modalities for delivery of patient care

**Fig. 4:**
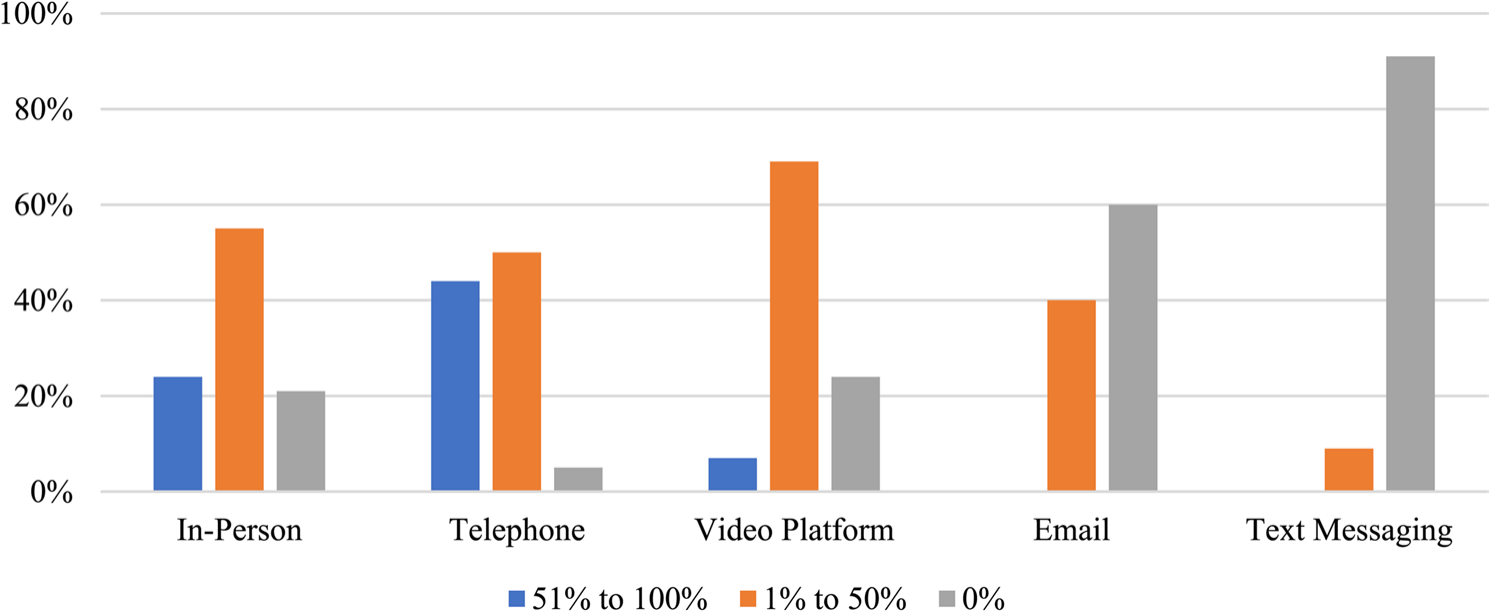
Actual modalities used by respondents to deliver patient care within the previous month (*n* = 131)

**Fig. 5:**
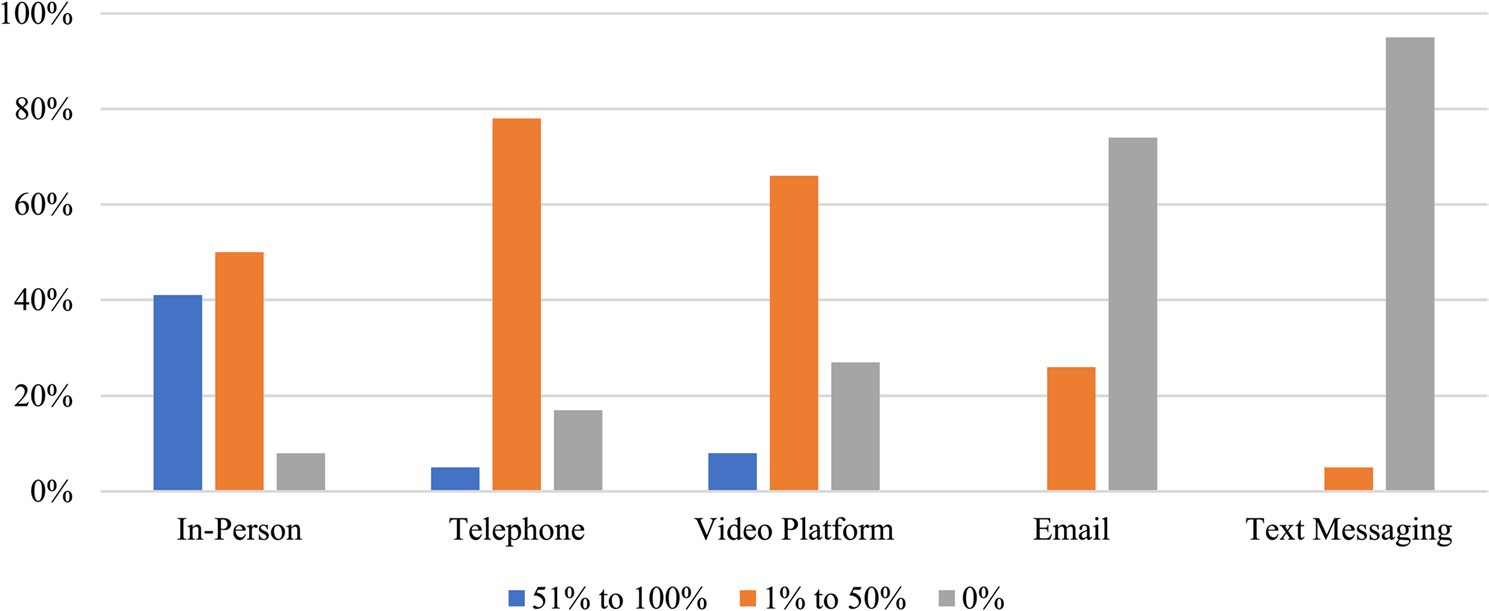
Preferred method for delivering patient care (*n* = 131)

Figure 4 highlights the *actual* modalities respondents used to deliver patient care within the previous month of completing the survey. Alternatively, Fig. 5 highlights respondents *preferred* modality of choice for the delivery of patient care.

## Discussion

This study provides critical insights into social work practice in primary care teams, with social workers clearly providing expanded access to a range of comprehensive health and mental health services for a broad scope of patient issues. Our study demonstrates that social workers are experts with broad and adaptable skillsets that align with the context of primary care, contributing to the diverse range of patient needs that emerge in primary care. Unsurprisingly, most social workers who completed this survey had graduate-level university training as a requirement for their employment in primary care. Each province in Canada has implemented legislation and created regulatory bodies to oversee the social work profession in line with that legislation [15]. For that reason, the 8% of respondents who indicated that registration was not a requirement for their position would likely have been in positions that did not use “social worker” as the title (i.e. mental health counsellor, management, etc.).

The demographics of our study participants provides insight into the nature of recruitment and retention of social workers in primary care. 57% of participants in our study had been in their positions for less than five years, with substantially less (15%) being in these positions for five to eight years. Our sample’s median age range (41–55), consistent with the median Canadian social worker age [35], indicates that this is not due to a preponderance of new graduates amongst our participants. Rather, our reported position durations are consistent with the two-to-five-year duration reported elsewhere for Ontarian FHT social workers [10, 36]. Notably, a notable minority (23%) of participants indicated they had been in their positions for over eight years. Determining what contributed to these social workers remaining within their organizations for almost half of the lifetime of FHTs is an important area for further study given the current recruitment and retention challenges facing healthcare [37, 38].

What was somewhat surprising in the results of our study was that the emphasis on addressing the social determinants was not as high as expected; for instance, only 23% of practitioners routinely addressed housing security. In contrast, an earlier study in Toronto [39] identified that social workers in major hospitals dedicated substantial portions of their work time to addressing social determinants, notably in ensuring access to healthcare services (92% of their time) and addressing housing concerns (72% of their time). The primary care social workers in our study, however, emphasized mental health as a key focal area of their clinical practice. Given that social determinants are directly entwined with mental health [40], social workers might be addressing the range of social and structural factors that are concerns for their patients within encounters specified as mental health.

Our results, however, suggest that there may be room for improvement in structuring the social work role in a way that provides greater efforts to explicitly address social determinants within primary care contexts given the recent urgent calls to strengthen social work and social prescribing in primary care [41–43]. In primary care, the social work role appears to be structured in a way that seems similar to traditional counselling formats with sequential one-hour appointments. There may be opportunity to reexamine approaches and length of patient appointments to further facilitate comprehensive care for complex patient care needs [43, 44]. Explicitly training social workers in primary care teams to routinely integrate social determinants of health in assessment, prevention, and treatment is recommended [45].

Our study results suggest that social workers are collaborative members of primary care teams which is unsurprising considering that social workers are trained for team process, group facilitation, and system navigation [24]. Social workers involved in this study were frequently engaged in collaborating with members of their primary care team and with those from other health and social care sectors. It is important to highlight that over a third of participants offered care to patients who were not rostered with their primary care clinic.

Social workers in our study demonstrated using a variety of modalities for connecting with their clients and colleagues. While reports from earlier in the pandemic [9, 21] indicated discomfort with virtual care technologies, the majority of our participants indicated comfort and a willingness to use virtual technologies post-pandemic. With current policy initiatives striving to enhance use of virtual care and digital tools in primary care [5], it is imperative for social workers to enhance their own capacity for using such technologies in practice in a way that aligns with patients needs. It is unsurprising, however, that social workers in our study indicated a preference for in-person interactions for collaboration with team members.

Collaborative work environments that enable a provider to meaningfully fulfil their role has been shown to improve primary care providers’ well-being and overall team functioning [36, 46, 47]. Existing guidelines to help primary care leaders determine how to structure social work roles in primary care are minimal [19]. Currently, there are no best practice guidelines on the recommended number of patients to be seen in a day or a given period. In our study, most social workers recommended that no more than five patients be seen for individual appointments in a day. Because of the emphasis on counselling in one-hour time-blocks versus system navigation and social prescribing, it is no wonder that social workers are recommending five patients per day for their caseload. Given that patients considered more complex in primary care tend to be those with needs related to mental health, social determinants of health, and other social vulnerabilities [48] – patient complexity inevitably is a part of social work practice in primary care and needs to be considered when developing guidelines for caseload numbers.

In terms of performance metrics, measures appear to emphasize the number of patient encounters a social worker has which does not adequately capture the quality of care nor impact of social workers’ interventions. Although volume counts may be valuable for illustrating access, volume alone does not capture the extent that patient complexity is being considered within social workers’ workloads [49]. Our study also provides guidance to social work educators on key areas to highlight in training. For example, general and specialized assessments were a frequent focus of social workers’ practice in primary care which suggests the need for social work educators to emphasize adequate preparation for such activities in their training. Importantly, our study also demonstrates that there is vast opportunity to bolster involvement of experienced primary care social workers in the education and training of students considering that over half of the study respondents had no such involvement.

### Strengths and limitations

There are several strengths related to this study. The study sample included geographical representation of social workers across the province of Ontario spanning the West, East, Central, Toronto, and North health regions. Another strength of the study sample was that social workers representation spanned the various types of interprofessional primary care models found across Ontario. Gaining an understanding of social workers’ clinical practice across the various models is useful for leaders and health system planners. However, there are also several limitations. The use of a survey to answer our research questions meant that the range of responses are limited to categories within the survey itself. As a cross-sectional, descriptive survey that captures a moment in time, it is difficult to make any causal inferences with our findings. This study only includes social workers in primary care teams in Ontario, Canada which may not be reflective of all jurisdictions. The recruitment method also lends to a biased sample as a specific type of social worker may be choosing to participate in our study. This means that we may be missing social workers who share characteristics that lead them to opt out from the survey and thus, skewing our findings.

## Conclusion

Social work has a crucial role in team-based primary care by providing a wide range of services to support the health, mental health, and social well-being of patients across Ontario. There was a large emphasis on addressing mental health needs of patients in our study, although there is a possibility to explicitly attend integrate the social determinants of health into care. Collaborating with other healthcare providers, social workers engage in a variety of practice activities from clinical engagement to community involvement to consultations. Recognizing that communities continue to shift and evolve, it will be crucial to regularly review the delivery and scope of social work practice to ensure it continues to align with patient needs. Social workers have also adapted to the changing demands of communities, offering services through different modalities and offering assistance to non-rostered patients. At the same time, developing best practices for social work at a structural and organizational perspective, with leadership from social workers, will further influence the optimization of care. As one of the largest health care providers in primary care, social work is a vital contributor in supporting and delivering effective, accessible, and patient-centred primary care.

## Supplementary Information

Supplementary Material 1.

## Data Availability

The datasets used and/or analyzed during the current study are available from the corresponding author on reasonable request.

## References

[1] Kiran T. Keeping the front door open: ensuring access to primary care for all in Canada. CMAJ. 2022;194(48):E1655–6. 10.1503/cmaj.221563. 36511860 10.1503/cmaj.221563 PMC9828980

[2] Feryn N, De Corte J, Roose R. Interprofessional primary care practice including social workers: exploring the experiences of patients in vulnerable situations. J Interp Care. 2022;36(6):793–800. 10.1080/13561820.2021.2015302. 10.1080/13561820.2021.2015302 35050834

[3] Peduzzi M, Aguiar C, Lima AMV, Montanari P, Leonello VM, de Oliveira MR. Expansion of the interprofessional clinical practice of primary care nurses. Revista Brasileira De Enfermagem. 2019;72(Suppl 1):114–21. 10.1590/0034-7167-2017-0759. 30942352 10.1590/0034-7167-2017-0759

[4] Glazier RH. Our role in making the Canadian health care system one of the world’s best: how family medicine and primary care can transform—and bring the rest of the system with Us. Can Fam Phys. 2023;69(1):11. 10.46747/cfp.690111. 10.46747/cfp.690111 PMC9873296 36693751

[5] Government of Ontario. Ontario’s primary care action plan. Connecting every person in Ontario to primary care. Ontario: Government of Ontario; 2025. Available from https://www.ontario.ca/files/2025-01/moh-ontario-primary-care-action-plan-overview-2025-01-27.pdf. Cited 2025 Feb 07.

[6] Starfield B, Shi L, Macinko J. Contribution of primary care to health systems and health. Mil Q. 2005;83(3):457–502. 10.1111/j.1468-0009.2005.00409.x. 10.1111/j.1468-0009.2005.00409.x PMC2690145 16202000

[7] Hutchison B, Levesque JF, Strumpf E, Coyle N. Primary health care in canada: systems in motion. Mil Q. 2011;89:256–88. 10.1111/j.1468-0009.2011.00628.x. 10.1111/j.1468-0009.2011.00628.x PMC3142339 21676023

[8] College of Family Physicians of Canada. A new vision for Canada: Family practice - the patient’s medical home. Mississagua: College of Family Physicians; 2019. Available from: https://patientsmedicalhome.ca/files/uploads/PMH_VISION2019_ENG_WEB_2.pdf. Cited 2025 Feb 07.

[9] Donnelly C, Ashcroft R, Bobbette N, Mills C, Mofina A, Tran T, et al. Interprofessional primary care during COVID-19: A survey of the provider perspective. BMC Fam Pract. 2021;22:31. 10.1186/s12875-020-01366-9. 33535973 10.1186/s12875-020-01366-9 PMC7857097

[10] Ashcroft R, McMillan C, Ambrose-Miller W, McKee R, Brown JB. The emerging role of social work in primary health care: A survey of social workers in Ontario family health teams. Health Soc Work. 2018;43(2):109–17. 10.1093/hsw/hly003. 29490042 10.1093/hsw/hly003

[11] Glazier R, Zagorski B, Rayner J. Comparison of primary care models in Ontario by Demographics, Case Mix and Emergency Department Use, 2008/09 to 2009/10. ICES investigative report. Toronto: Institute of Clinical and Evaluative Sciences; 2012. Available from: https://www.ices.on.ca/wp-content/uploads/2023/06/Full-report-51.pdf. Cited 2025 Feb 07.

[12] United States Department of Labor Bureau of Labor Statistics. Occupational Employment and Wages [Internet]. United States Department of Labor Bureau of Labor Statistics; 2024. Available from https://www.bls.gov/oes/current/oes211022.htm. Cited 2025 Feb 07.

[13] Zerden LD, Lombardi BM, Richman EL. Social workers on the interprofessional integrated team: elements of team integration and barriers to practice. J Interp Educ Pract. 2019;17:100286. 10.1016/j.xjep.2019.100286.

[14] Ashcroft R, Donnelly C, Dancey M, Gill S, Lam S, Kourgiantakis T, et al. Primary care teams’ experiences of delivering mental health care during the COVID-19 pandemic: a qualitative study. BMC Fam Pract. 2021;22:1–12. 10.1186/s12875-021-01496-8. 34210284 10.1186/s12875-021-01496-8 PMC8248293

[15] Kourgiantakis T, Ashcroft R, Mohamud F, Benedict A, Lee E, Craig S, et al. Clinical social work practice in canada: A critical examination of regulation. RSWP. 2023;33(1):15–28. 10.1177/104973152211094.

[16] Sur D, Ashcroft R, Adamson K, Tanner N, Webb J, Mohamud F, et al. Examining diagnosis as a component of social workers’ scope of practice: A scoping review. Clin Soc Work. 2023;51:12–23. 10.1007/s10615-022-00838-y.

[17] Stanhope V, Videka L, Thorning H, McKay M. Moving toward integrated health: an opportunity for social work. Soc Work Health Care. 2015;5383–407. 10.1080/00981389.2015.1025122. 10.1080/00981389.2015.1025122 25985284

[18] Canadian Institute for Health Information. Social Workers. About Social Workers. Canadian Institute for Health Information. Available from: https://www.cihi.ca/en/social-workers. Cited 2025 Feb 07.

[19] Ashcroft R, Sheffield P, Adamson K, Phelps F, Webber G, Walsh B, et al. Scoping review of social workers’ professional roles in primary care. BMJ Open. 2024;14(12):e090527. 10.1136/bmjopen-2024-090527. 39740939 10.1136/bmjopen-2024-090527 PMC11749816

[20] McGregor J, Mercer SW, Harris FM. Health benefits of primary care social work for adults with complex health and social needs: a systematic review. Health Soc Care Community. 2018;26(1):1–13. 10.1111/hsc.12337. 27059167 10.1111/hsc.12337

[21] Ashcroft R, Sur D, Greenblatt A, Donahue P. The impact of the COVID-19 pandemic on social workers at the frontlines. Br J Soc Work. 2022;52(3):1724–46. 10.1093/bjsw/bcab158.

[22] Horevitz E, Manoleas P. Professional competencies and training needs of professional social workers in integrated behavioral health in primary care. Soc Work Health Care. 2013;52(8):752–87. 10.1080/00981389.2013.791362. 24028739 10.1080/00981389.2013.791362

[23] Steketee G, Ross AM, Wachman MK. Health outcomes and costs of social work services: A systematic review. Am J Public Health. 2017;107(S3):S256–66. 10.2105/AJPH.2017.304004. 29236534 10.2105/AJPH.2017.304004 PMC5731071

[24] Ashcroft R, Feryn N, Lam S, Hussain A, Donnelly C, Mehta K, et al. Social workers’ formal and informal leadership roles in interprofessional primary care teams. Healthc Manage Forum. 2023;36(5):304–10. 10.1177/08404704231184582. 37392058 10.1177/08404704231184582 PMC10445548

[25] Dihn T, Bounajm F. Improving Primary Health Care through Collaboration: Measuring the Missed Opportunity [Internet]. The Conference Board of Canada; 2013 June [cited 2025 Feb 07]. Available from https://professionals.wrha.mb.ca/old/professionals/collaborativecare/files/IPHCTC-Briefing3.pdfhttp://www.integrationresources.ca/wordpress/wp-content/uploads/2013/09/D43_PrimaryHealthCare_Briefing_3.pdf.

[26] Donabedian A. The quality of care. How can it be assessed? JAMA. 1988;260(12):1743–8. 10.1001/jama.260.12.1743. 3045356 10.1001/jama.260.12.1743

[27] Government of Ontario. Ontario Demographic Quarterly: highlights of second quarter. 2024. Available from https://www.ontario.ca/page/ontario-demographic-quarterly-highlights-second-quarter. Cited 2025 Feb 07.

[28] Rudoler D, Laporte A, Barnsley J, Glazier RH, Deber RB. Paying for primary care: a cross-sectional analysis of cost and morbidity distributions across primary care payment models in Ontario Canada. Soc Sci Med. 2015;124:18–28. 10.1016/j.socscimed.2014.11.001. 25461858 10.1016/j.socscimed.2014.11.001

[29] Canadian Institute for Health Information (CIHI). Primary Care Clients at Ontario Community Health Centres: Characteristics and Service Use. Ottawa, ON: CIHI. 2024. Available from: https://www.cihi.ca/sites/default/files/document/primary-care-clients-ontario-chc-characteristics-service-use-report-en.pdf. Cited 2025 Feb 07.

[30] Nurse Practitioners’ Association of Ontario. Nurse practitioner-led clinics. Nurse Practitioners’ Assocation of Ontario; 2020. Available from: https://npao.org/nplcs/. Cited 2025 Feb 07.

[31] Indigenous Primary Health Care Council. Find a provider - Indigenous Primary Health Care Council. Indigenous Primary Health Care Council; 2023. Available from: https://iphcc.ca/myhealth/. Cited 2025 Feb 07.

[32] Albudaiwi D. Surveys, advantages, and disadvantages. In: Allen M, editor. The SAGE encyclopedia of communication research methods. Thousand Oaks: SAGE; 2018. pp. 1735–6.

[33] Donnelly CA, Leclair LL, Wener PF, Hand CL, Letts LJ. Occupational therapy in primary care: results from a National survey. Can J Occup Ther. 2016;83(3):135–42. 10.1177/0008417416637186. 27074910 10.1177/0008417416637186

[34] Drennan J. Cognitive interviewing: verbal data in the design and pretesting of questionnaires. J Adv Nurs. 42(1); 57–63. 10.1046/j.1365-2648.2003.02579.x 10.1046/j.1365-2648.2003.02579.x 12641812

[35] Mirshahi R, Baczkowska M. Social Workers. In I.Bourgeault, editor, Introduction to Health Occupations in Canada. Ottawa: Canadian Health Workforce Partners. 2023:451–470.

[36] Brown JB, Ryan BL. Processes that influence the evolution of family health teams. Can Fam Physician. 2018;64(6):e283–89. 29898949 PMC5999255

[37] Murphy GT, Sampalli T, Carson A, Embrett M, Sim M, Chamberland-Rowe C, et al. Impact of the COVID-19 pandemic on the Canadian healthcare workforce: a rapid evidence synthesis of key considerations, lessons learned, and promising practices to address the healthcare workforce crisis. Health Res Policy Syst. 2025;23:132. 10.1186/s12961-025-01395-9. 41088358 10.1186/s12961-025-01395-9 PMC12522264

[38] Okpalauwaekwe U, MacPhee BK, Balezantis L, Mahani A, Zarzeczny, et al. Facilitators, barriers, and priorities for enhancing primary care recruitment and retention in Saskatchewan, Canada. BMC Prim Care. 2025;26:373. 10.1186/s12875-025-03049-9. 41266998 10.1186/s12875-025-03049-9 PMC12636174

[39] Craig SL, Bejan R, Muskat B. Making the invisible visible: are health social workers addressing the social determinants of health? Soc Work Health Care. 2013;52(4):311–31. 10.1080/00981389.2013.764379. 23581836 10.1080/00981389.2013.764379

[40] Kirkbride JB, Anglin DM, Colman I, Dykxhoorn J, Jones PB, Patalay P, et al. The social determinants of mental health and disorder: evidence, prevention and recommendations. World Psy. 2024;23(1):58–90. 10.1002/wps.21160. 10.1002/wps.21160 PMC10786006 38214615

[41] Chng NR, Hawkins K, Fitzpatrick B, O’Donnell CA, Mackenzie M, Wyke S, et al. Implementing social prescribing in primary care in areas of high socioeconomic deprivation: process evaluation of the ‘Deep end’ community links worker programme. Br J Gen Pract. 2021;71(713):e920. 10.3399/BJGP.2020.1153. 10.3399/BJGP.2020.1153 PMC8463130 34019479

[42] Nowak DA, Mulligan K. Social prescribing: A call to action. Can Fam Phys. 2021;67(2):88–91. 10.46747/cfp.67028. 10.46747/cfp.670288 PMC8324130 33608356

[43] Swanson KM, Matulis JC, McCoy RG. Association between primary care appointment lengths and subsequent ambulatory reassessment, emergency department care, and hospitalization: a cohort study. BMC Prim Care. 2022;23(1):39. 10.1186/s12875-022-01644-8. 35249539 10.1186/s12875-022-01644-8 PMC8900401

[44] Campbell SM, Roland MO, Buetow SA. Defining quality of care. Soc Sci Med. 2000;51:1611–25. 10.1016/S0277-9536(00)00057-5. 11072882 10.1016/s0277-9536(00)00057-5

[45] Pinto A, Bloch G. Framework for building primary care capacity to address the social determinants of health. Can Fam Phys. 2017;63: e476-e482. Available from: https://www.cfp.ca/content/cfp/63/11/e476.full.pdf. PMC5685463 29138172

[46] LinzerM, Manwell LB, Williams ES, Bobula JA, Brown RL, Varkey AB, et al. Working conditions in primary care: physician reactions and care quality. Ann Intern Med. 2009;151(1):28–36. 10.7326/0003-4819-151-1-200907070-00006. 19581644 10.7326/0003-4819-151-1-200907070-00006

[47] Connolly SL, Ferris SD, Azario RP, Miller CJ. Patient and provider attitudes toward video and phone telemental health care during the COVID-19 pandemic: A systematic review. Clin Psychol: Sci Pract. 2024;31(4):488–503. 10.1037/cps0000226. 10.1037/cps0000226 PMC11931715 40129719

[48] Loeb DF, Binswanger IA, Candrian C, Bayliss EA. Primary care physician insights into a typology of the complex patient in primary care. Ann Fam Med. 2015;13(5):451–5. 10.1370/afm.1840. 26371266 10.1370/afm.1840 PMC4569453

[49] Ashcroft R. Inadequate performance measures affecting practices, organizations and outcomes of ontario’s family health teams. Healthc Policy. 2014;10(1):86–96. 25410698 PMC4253898

