# Supplementary material for: Enhancing comprehensive in primary care: results of a cross-sectional survey of primary care social workers in Ontario, Canada
Source: BMC Prim Care. 2026 Feb 27;27:116. doi: 10.1186/s12875-026-03226-4 (PMC13049781; doi:10.1186/s12875-026-03226-4)
Supplement: Supplementary file 1 — Supplementary Material 1. [file 12875_2026_3226_MOESM1_ESM.pdf]

FACTOR-INWENTASH
FACULTY OF SOCIAL WORK
UNIVERSITY OF TORONTO

Consent

Social Workers in Primary Care 2022

Study Information and Consent

Purpose of the Study

The primary purpose of this study is to understand the structure of **social work practice** in primary care settings across Ontario during the COVID-19 pandemic. We are inviting social workers working in primary care settings across Ontario to share their experiences since the start of COVID-19.

Study Participants

This study is aimed at social workers working in primary care settings in Ontario. Social workers in primary care settings include:

- Individuals who are working in designated social work roles in primary care; Individuals formally trained in social work (e.g. BSW/MSW) and practicing as a social worker in primary care in a non-specified social work role (e.g. mental health counsellor, registered psychotherapist).

Study Procedures

If you decide to participate, you will complete a survey that consists of 45 questions and will take approximately 20 minutes to finish.

Risks

Although there are no physical or psychological/ emotional risks expected, we realize that you may experience minor discomfort from sharing your experiences in this survey. There will be no identifying personal information asked from participants in this survey.

Benefits

We do not expect direct benefit for you from participation in this study. However, your participation will provide information that will help to better understand the role and influence of social workers within primary care

settings in Ontario.

Voluntary Participation

Your decision to participate in this study is voluntary. You may choose to withdraw from the study at any time even if you sign this consent. Should you wish to withdraw, please contact Rachelle Ashcroft at rachelle.ashcroft@utoronto.ca, and state that you would like your data removed from the survey and deleted.

Rights and Confidentiality

Any information that could be identifying for participants will be kept strictly confidential and anonymous. Neither your email, nor any other personal identifier will be used in any reports or publications arising from this study. In addition, all data records will be kept on a secure server at the University of Toronto, Factor-Inwentash Faculty of Social Work. This information will be encrypted, password protected and will only be accessible by the research team members for the purpose of this study.

Dissemination of Results

The results of this study will be used for research presentations, conferences, and publications. Should you have any questions about the study or wish to receive a copy of the results of this study, please contact Rachelle

Ashcroft at rachelle.ashcroft@utoronto.ca.

Additional Information
This study has been reviewed and received ethics clearance through the University of Toronto, Office of Research Ethics (RIS #42283). You may also contact the University of Toronto, Office of Research Ethics for questions about your rights as a research participant at ethics.review@utoronto.ca or 416-946-3273. The final decision about participation is yours. Your participation is important to the study. We hope you will agree to take part.

Consent:
- I understand that my participation in this study is voluntary
- I understand that all data will be de-identified, and there will be no identifying information on any disseminated work
- I understand that we will keep all data secure to protect confidentiality for all participants
- I understand that I may withdraw consent from the study at any time by emailing rachelle.ashcroft@utoronto.ca

Research study approved by the University of Toronto

(RIS #42283)

Do you provide consent to participate in this study?

- ○ Yes, I provide consent to participate in this study
- ○ No, I do not provide consent to participate in this study

Location of Practice

Getting to Know Location of Practice

Within your primary care role, which Ontario Health Region do you work in?

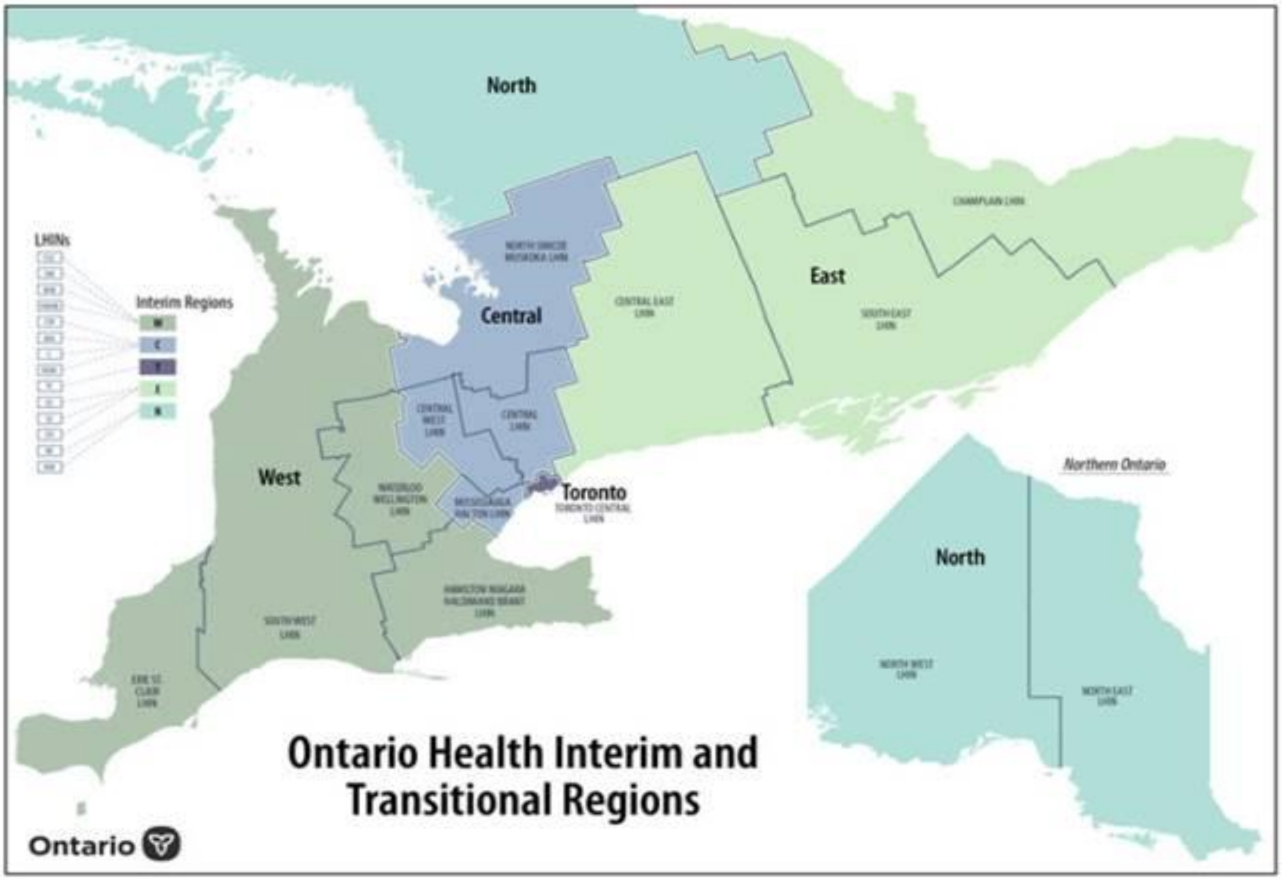

- North
- East
- West
- Central
- Toronto

Which primary care model do you currently work in?

- ○ Family Health Team (FHT)
- ○ Community Health Centre (CHC)
- ○ Family Physician Office (FPO)
- ○ Nurse Practitioner Led Clinic (NPLC)
- ○ Aboriginal Health Access Centre (AHAC)
- ○ [ ] Other (please specify)

Job Information

Getting to Know Your Background as a Social Worker in Primary Care

What is your formal job title? (check all that apply)

- ☐ Social Worker
- ☐ Mental Health Counsellor
- ☐ Registered Psychotherapist
- ☐ [ ] Other, please specify:

What is your current employment status in primary care? (If more than one, please select the most applicable one)

- ○ Permanent full-time (>36 hours/week for one employer)
- ○ Permanent part-time (18-35 hours/week for one employer)
- ○ Permanent part-time (1-17 hours/week for one employer)
- ○ Temporary full-time (e.g., maternity leave coverage)
- ○ Temporary part-time
- ○ Casual (no set number of hours)
- ○ [ ] Other, please specify:

What is your educational background? (check all that apply)

- ☐ Bachelor of Social Work (BSW)
- ☐ Master of Social Work (MSW)
- ☐ Doctorate of Philosophy in Social Work (PhD)
- ☐ Social Service Worker Diploma (SSW)
- ☐ [ ] Other, please specify:

What is the REQUIRED educational background for the position you currently hold? (check all that apply)

- ☐ Bachelor of Social Work (BSW)
- ☐ Master of Social Work (MSW)
- ☐ Doctorate of Philosophy in Social Work (PhD)
- ☐ Social Service Worker Diploma (SSW)
- ☐ [ ] Other, please specify:

How many years have you been practicing social work?

- ○ < 1 year
- ○ 1 - 5 years
- ○ 6 - 10 years
- ○ 11 - 15 years
- ○ 16 - 20 years
- ○ > 20 years

How long have you worked in your current position?

- ○ < 1 year
- ○ 1 - 2 years
- ○ 3 - 4 years
- ○ 5 - 6 years
- ○ 7 - 8 years
- ○ > 8 years

Is registration with the Ontario College of Social Workers and Social Service Workers a requirement for your job in primary care?

- Yes
- No
- Unsure

Primary Care Context

Getting to Know Your Primary Care Context

What is the total number of social workers (including yourself) hired by your primary care organization (this includes social workers working at other sites of the same organization)?

- 1
- 2 - 3
- 4 - 5
- 6 - 7
- 8 - 9
- > 9

At your physical workplace, how many social workers in total (including you) are **CO-LOCATED** within the same primary care organization?

- 1
- 2 - 3
- 4 - 5
- 6 - 7
- 8 - 9
- > 9

What other service providers comprise your primary care team? (check all that apply)

- ☐ Family Physician
- ☐ Psychiatrist
- ☐ Nurse Practitioner or Advanced Practice Nurse
- ☐ Registered Nurse
- ☐ Occupational Therapist
- ☐ Physiotherapist
- ☐ Physician Assistant
- ☐ Social Worker
- ☐ Mental Health Counsellor
- ☐ Psychologist
- ☐ Registered Dietitian
- ☐ Pharmacist
- ☐ __________ Other, please specify:

Of these other team members, how often do you directly interact and communicate with the following team members about a clinical situation?

Frequency of Collaboration

Pr
r

	Daily	Weekly	Occasionally	Never
Family Physician	○	○	○	○
Psychiatrist	○	○	○	○
Nurse Practitioner or Advance Practice Nurse	○	○	○	○
Registered Nurse	○	○	○	○
Occupational Therapist	○	○	○	○
Physiotherapist	○	○	○	○
Physician Assistant	○	○	○	○
Social Worker	○	○	○	○
Mental Health Counsellor	○	○	○	○
Psychologist	○	○	○	○
Registered Dietitian	○	○	○	○
Pharmacist	○	○	○	○
Other, please specify: ______	○	○	○	○

Thinking about the last 30 days, what methods did you use to interact and communicate with your team? (check all that apply)

- ☐ Informal and unplanned in-person discussions
- ☐ Formal/scheduled in-person discussions
- ☐ Video meetings (e.x., Zoom, Microsoft Teams)
- ☐ Telephone calls
- ☐ EMR communications
- ☐ Email
- ☐ Text messaging
- ☐ Social media
- ☐ __________ Other, please specify:

Post COVID-19, what do you prefer as the main modes of communication between yourself and other team members? (check all that apply)

- ☐ Informal and unplanned in-person discussions
- ☐ Formal/scheduled in-person discussions
- ☐ Video meetings (e.x., Zoom, Microsoft Teams)
- ☐ Telephone calls
- ☐ EMR communications
- ☐ Email
- ☐ Text messaging
- ☐ Social media
- ☐ [ ] Other, please specify:

Structure of Practice

Understanding the Structure of your Practice

Please identify the most common ways patients are referred to your services. (check all that apply)

- ☐ Referral from family physician or nurse practitioner
- ☐ Referral from other interprofessional health provider
- ☐ First-contact (e.g. patients directly book into your services as needed without a referral)
- ☐ Other, please specify: ______

Typically, how many patients do you see for individual appointments in a typical 8-hour workday?

- ○ 1 - 5
- ○ 6 - 10
- ○ 11 - 15
- ○ > 15
- ○ I do not provide direct patient care
- ○ Other, please specify: ______

Typically, how many patients do you see for individual appointments in a week?

- 1 - 5
- 6 - 10
- 11 - 15
- 16 - 20
- 21 - 25
- 26 - 30
- > 30
- I do not provide direct patient care
- Other, please specify: ________

Typically, how long do you schedule a follow-up session with an individual patient?

- Less than half an hour (<30 mins)
- Between half an hour to an hour (30 - 60 mins)
- Between 1 to 2 hours (61 - 120 mins)
- Between 2 to 3 hours (121 mins to 180 mins)
- Over 3 hours (>181 mins)
- I do not provide direct patient care
- Other, please specify: ________

What is the typical wait time for a patient to see you for an **URGENT** referral?

- Same day
- 1 week
- 2 weeks
- 3 weeks
- 4 weeks
- 5 or more weeks
- I do not receive or provide services for urgent referrals (e.g., due to long waitlist, high caseload, not part of duties etc.)
- I do not provide direct patient care

What is the typical wait time for a patient to see you for a **NON-URGENT** referral?

- Same day
- 1 week
- 2 weeks
- 3 weeks
- 4 weeks
- 5 or more weeks
- I do not provide direct patient care

Are you providing care to patients outside of your practice’s patient roster?

- Yes
- No
- Not applicable

Please describe your care to patients outside of your practice's patient roster, for example, where does this happen, how often, and how much time do you offer?

What performance metrics are being used to specifically track your work? (e.x., self-reported stats, data in electronic medical records, etc.).

Do you think that the performance metrics currently used to track social work's contribution to patient care are adequate?

- ○ Yes, please explain:
- ○ No, please explain:
- ○ Unsure or Not Applicable

Improving Structure

Your Recommendations for Improving the Structure of Practice

For the next set of questions, we want you to think about how you might improve the structure of your practice.

What is the maximum number of patients you think a social worker in primary care should see in a typical 8-hour workday?

- 1 - 5
- 6 - 10
- 11 - 15
- Other, please specify:

For social workers in primary care, what do you think is an optimal length of appointment time for a follow-up session with an individual patient?

- Less than half an hour (<30 mins)
- Between half an hour to an hour (30 - 60 mins)
- Between 1 to 2 hours (61 - 120 mins)
- Between 2 to 3 hours (121 mins to 180 mins)
- Over 3 hours (>181 mins)
- Other, please specify:

Are there changes you would like to see to the referral process for social work?

- ○ Yes, please explain: ________
- ○ No

Please describe any other changes you would make to improve the structure of your practice.

____

Activities

Understanding Your Role and Practice Activities

In the last month, please estimate what percentage (%) of your practice has been directed to each of the following:

In Percentages, please ensure it adds to 100%

0 10 20 30 40 50 60 70 80 90 100

Individual Counselling

Couple and/or Family Counselling

Groups

Other Direct Patient Care Activities

Indirect Patient-Care Activities

Others, please specify:

Total: **0**

Which patient populations do you most frequently work with? (check all that apply)

- ☐ Older adults (> 64)
- ☐ Adults (30-64)
- ☐ Young Adults (19-29)
- ☐ Youth (13-18)
- ☐ Children (under 13)

What types of patient groups do you offer and/or lead?

In the last month, please estimate how frequently your social work practice has been directly focused on each of the following practice areas:

	Daily	Weekly	Monthly	Rarely	Never
Mental health	○	○	○	○	○
Addictions	○	○	○	○	○
Chronic disease management	○	○	○	○	○
Memory clinic and/or other neurological issues	○	○	○	○	○
Palliative care	○	○	○	○	○
Grief & bereavement (not palliative care related)	○	○	○	○	○
Trans-specific and gender affirming care	○	○	○	○	○
Geriatrics and aging	○	○	○	○	○
Children and youth	○	○	○	○	○
Parenting issues	○	○	○	○	○
Financial stressors, social assistance	○	○	○	○	○
Housing insecurity	○	○	○	○	○
Legal issues	○	○	○	○	○
Other, please specify: [ ]	○	○	○	○	○

In the last month, please estimate how frequently you engaged in the following practice activities:

	Daily	Weekly	Monthly	Rarely	Never
General psychosocial assessments	○	○	○	○	○
Formal assessments using a tool (e.g., PHQ9)	○	○	○	○	○
Direct counselling/therapy	○	○	○	○	○
Leading groups	○	○	○	○	○
Case management	○	○	○	○	○
Systems navigation and accessing community resources	○	○	○	○	○
Educating and training students	○	○	○	○	○
Providing consultation to healthcare providers WITHIN your team (related to patient care)	○	○	○	○	○
Providing consultation to healthcare providers OUTSIDE of your team (related to patient care)	○	○	○	○	○
Documentation	○	○	○	○	○
Other, please specify: ______	○	○	○	○	○

In the last month, how often (%) did you use the following modalities to deliver patient care?

In Percentages, please ensure it adds to 100%

	0 10 20 30 40 50 60 70 80 90 100	
In-person		0
Video platform		0
Telephone		0
Email		0
Text messaging		0
Other		0
**Total:**		**0**

Post-COVID 19, how often (%) would you prefer to continue providing services through these modalities?

	In Percentages, please ensure it adds to 100% (0 10 20 30 40 50 60 70 80 90 100)	
In-person		0
Video platform		0
Telephone		0
Email		0
Text messaging		0
Other		0
**Total:**		**0**

Leadership

Social Work Leadership in Primary Care

For the following questions, we want to learn the various ways that social workers are providing formal and informal leadership in primary care.

How do you currently demonstrate leadership skills in your social work role?

- ○ I hold a formal leadership role on my team
- ○ I informally provide leadership to my team
- ○ I do not currently demonstrate leadership in my role

In your current role in primary care, what types of leadership activities do you engage in? (check all that apply)

- ☐ Engage in program development and/or program evaluation activities
- ☐ Quality improvement
- ☐ Train, mentor, or supervise team members
- ☐ Support team collaboration and team dynamics
- ☐ Organize and/or lead team meetings
- ☐ Provide consultation to other disciplines about patient care case
- ☐ Directly influence the decision-making processes within my team
- ☐ Facilitate partnerships with services or other providers outside of my primary care organization
- ☐ Act as a representative for local stakeholder groups (e.g. Ontario Health Team Stakeholder group, AFHTO)
- ☐ [ ] Other, please specify:

What are the various ways that you have provided leadership to your primary care team during the COVID-19 pandemic?

- ☐ Participating in formal COVID-19 response and planning
- ☐ Planning and implementing transitioning to virtual care
- ☐ Facilitating patient access
- ☐ Implementing activities that addressed team well-being
- ☐ Implementing new innovations for patient care
- ☐ Helping to implement new vaccination clinics
- ☐ ________ Other, please specify:

Please rate your confidence in providing formal and/or informal leadership in your primary care team.

- ○ Very confident
- ○ Fairly confident
- ○ Not very confident
- ○ Not at all confident

What do you think supports your capacity to demonstrate leadership within your primary care team? (check all that apply)

- ☐ I received formal education or training on leadership
- ☐ I have received guidance and/or supervision that helped me to develop leadership skills
- ☐ I regularly have opportunity to display leadership skills in the team
- ☐ I have a personal interest in leadership opportunities or demonstrating leadership skills
- ☐ There are opportunities for social workers to hold formal leadership positions in primary care
- ☐ Other, please specify: ____________

What would further help strengthen your leadership capacity? (check all that apply)

- ☐ Receiving education or training on leadership skills for social workers
- ☐ Receiving oversight and/or supervision to develop leadership skills
- ☐ Receiving opportunities to display leadership skills on my team
- ☐ Developing confidence in my individual leadership skills
- ☐ Accessing opportunities for formal leadership positions for social workers
- ☐ I’m not interested in strengthening my leadership capacity
- ☐ Other, please specify:

Demographics

Demographics Questions

What is your age?

- 18 - 25
- 26 - 40
- 41 - 55
- 56 - 70
- > 70
- Prefer not to answer

What is your race and/or ethnicity? (check all that apply)

- ☐ First Nations
- ☐ Métis
- ☐ Inuk/Inuit
- ☐ Arab
- ☐ Black
- ☐ Latin American
- ☐ Chinese
- ☐ Filipino
- ☐ Japanese
- ☐ Korean
- ☐ South Asian (e.g., East Indian, Pakistani, Sri Lankan)
- ☐ Southeast Asian (e.g., Vietnamese, Cambodian, Laotian, Thai)
- ☐ West Asian (e.g., Iranian, Afghan)
- ☐ White/Caucasian
- ☐ Other, please specify: ____________
- ☐ Prefer not to answer

What is your gender?

- Male
- Female
- Non-binary
- Trans male
- Trans female
- Two-Spirit
- [ ] Other, please specify:
- Prefer not to answer

Participation in focus group

Future Focus Groups

We will be holding focus groups with social workers in the coming months to learn about recommendations on how to better support social work practice in primary care.

Would you like to be contacted about participation in a focus group on social workers in primary care?

- Yes
- No

Please provide us with your contact information:

Full Name

Email

Powered by Qualtrics
